# The content validity of the ANMS GCSI-DD in patients with idiopathic or diabetic gastroparesis

**DOI:** 10.1186/s41687-018-0081-2

**Published:** 2018-12-13

**Authors:** Dennis A. Revicki, Sara Lavoie, Rebecca M. Speck, Jorge Puelles, Braden Kuo, Michael Camilleri, Cristina Almansa, Henry P. Parkman

**Affiliations:** 1 Evidera, 7101 Wisconsin Avenue, Suite 1400, Bethesda, MD 20814 USA; 2 Takeda Pharmaceuticals, 61 Aldwych, London, WC2B 4AE UK; 3 Massachusetts General Hospital, 55 Fruit Street, Boston, MA 02114 USA; 4 Mayo Clinic, 200, 1st Street SW, Rochester, MN 55905 USA; 5 Takeda Pharmaceuticals, 35 Lansdowne Street, Cambridge, MA 02139 USA; 6 Temple University Hospital, 3401 N Broad Street, #1003, Philadelphia, PA 19140 USA

**Keywords:** Qualitative, Symptoms, HRQL, Patient-reported outcome, Content validity, Gastroparesis

## Abstract

**Background:**

The American Neurogastroenterology and Motility Society Gastroparesis Cardinal Symptom Index-Daily Diary (ANMS GCSI-DD) was developed to meet Food and Drug Administration (FDA) recommendations for patient-reported outcome (PRO) endpoints in gastroparesis studies, including therapeutic trials. The current version of the ANMS GCSI-DD contains five items pertaining to nausea, early satiety, post-prandial fullness, upper abdominal pain, and vomiting. The specific aims of this study were to determine if the appropriate symptoms are included in the ANMS GCSI-DD and to assess the content validity in patients with idiopathic (IG) and diabetic gastroparesis (DG).

**Methods:**

Patients diagnosed with IG or DG were recruited by five clinical sites in the United States for a cross-sectional, qualitative study involving one-on-one in-person concept elicitation and cognitive debriefing interviews. Concept elicitation included open-ended questions to elicit patients’ symptoms and impacts of gastroparesis, while cognitive debriefing was designed to assess the comprehensiveness of the ANMS GCSI-DD and clarity of the instructions, items, and response scales. The interviews were audio-recorded and transcribed. Transcripts were analyzed using a content analysis approach with ATLAS.ti.

**Results:**

Of 25 patients interviewed, 15 (60%) had IG and 10 (40%) DG. Mean age of the sample was 42.3 years (range: 20–70 years), and most patients were female (*n* = 19, 76%) and white (*n* = 19, 76%). During concept elicitation, patients endorsed the following signs and symptoms as relevant and important to their condition: early satiety (*n* = 25, 100%), post-prandial fullness (*n* = 25, 100%), nausea (*n* = 22, 88%), upper abdominal pain (*n* = 18, 72%), vomiting (*n* = 15, 60%), and bloating (*n* = 11, 44%). Many patients (*n* = 20, 80%) experienced day-to-day symptom change. During cognitive debriefing, patients confirmed the ANMS GCSI-DD content was comprehensive and reflective of their gastroparesis experience. Patients could easily select a response option and describe how they arrived at their answers. Overall, patients found the instrument’s instructions, recall period, items, and response options clear and understandable.

**Conclusions:**

The ANMS GCSI-DD was easily understood, found to contain the most important symptoms for patients with IG and DG, and no changes were recommended. Results support the content validity of the ANMS GCSI-DD for clinical trials and clinical care among IG or DG patients.

**Electronic supplementary material:**

The online version of this article (10.1186/s41687-018-0081-2) contains supplementary material, which is available to authorized users.

## Background

Gastroparesis is a symptomatic chronic disorder of the stomach characterized by delayed gastric emptying when no mechanical obstruction is present [3, 22]. Patients with gastroparesis often experience a variety of symptoms including early satiety, postprandial fullness, nausea, vomiting, and abdominal pain. Gastroparesis of unknown etiology (idiopathic gastroparesis; IG) accounts for the largest number of cases, but gastroparesis is also frequently associated with conditions such as diabetes (diabetic gastroparesis; DG). Gastroparesis may occur after various types of gastrointestinal surgeries, and is less frequently associated with other etiologies such as Parkinson’s disease, collagen vascular disorders, and internal pseudo-obstruction. Gastroparesis is associated with reduced quality of life and impaired day-to-day functioning [38]. Data from the Rochester Epidemiology Project indicate that the prevalence of diagnosed gastroparesis is 24.2/100,000 individuals [12], but the prevalence is likely underestimated given that a large group of people with gastroparesis-like symptoms have never had a gastric emptying (GE) test to confirm their diagnosis. A study using data from 450 patients showed that delayed GE was estimated to occur in 1.8% of community subjects [33]. If this is correct, the prevalence of gastroparesis in the United States (US) is estimated at 5 million to 10 million individuals (approximately 3% of the total population) and is approximately four times higher in women compared to men [13].

A symptom questionnaire, the Gastroparesis Cardinal Symptom Index (GCSI), was originally developed through patient interviews and was tested and validated in university-based clinical practices for quantifying symptoms in gastroparesis [28, 29]. The nine-symptom GCSI is based on three subscales (postprandial fullness/early satiety, nausea/vomiting, and bloating) and represents a subset of the longer 20-symptom Patient Assessment of Upper Gastrointestinal Disorders-Symptoms (PAGI-SYM). The GCSI and PAGI-SYM both ask patients to describe the severity of their symptoms over the last two weeks. The GCSI is the most frequently used symptom severity measure in gastroparesis clinical trials and other clinical studies [1, 2, 4–6, 9, 14, 16, 19, 21, 35, 36], including the NIH Gastroparesis Clinical Research Consortium Gastroparesis Registry study [10, 24].

The Food and Drug Administration (FDA) has been reevaluating symptom endpoints used in clinical trials of pharmaceutical agents for all disorders, including gastroparesis, and suggests using patient-reported outcome (PRO) measures to assess symptom endpoints. In July 2015, the FDA Gastroparesis Guidance for Industry was released; the guidance document contained recommendations for endpoints for gastroparesis studies [7]. A daily diary version of the GCSI, the American Neurogastroenterology and Motility Society Gastroparesis Cardinal Symptom Index-Daily Diary (ANMS GCSI-DD) was developed to meet these recommendations and is intended as a PRO measure used to assess symptom-based endpoints for new treatments in gastroparesis clinical trials [30, 31].

The ANMS GCSI-DD was developed by the ANMS GCSI-DD PRO Committee, consisting of representatives of ANMS interested in gastroparesis and the creator of the original GCSI questionnaire. The initial ANMS GCSI-DD version was based on the existing two-week GCSI questionnaire. The development of the diary has involved changing the instructions, recall period, and response scales; however, the item stems remain the same. In response to suggestions from clinical experts and the FDA, the diary was revised to inquire about symptoms of upper abdominal pain, which was not present in the original GCSI. In some patients, abdominal pain and/or discomfort can be an important symptom [10, 11], and has been used in other severity scores for gastroparesis [17, 27]. The upper abdominal pain item was based on the PAGI-SYM questionnaire, which has been previously tested and validated in patients with different gastrointestinal disorders [29, 32]. Suggestions from the FDA were also incorporated into the ANMS GCSI-DD including changing the vomiting assessment from severity to frequency. The ANMS GCSI-DD version used in the study reported herein is paper-based, uses a 24-h recall period, and consists of five symptoms: nausea, early satiety, post-prandial fullness, upper abdominal pain, and vomiting. Nausea, early satiety, post-prandial fullness, and upper abdominal pain are rated using a 5-point verbal rating scale, and the number of vomiting episodes over the last 24 h is recorded by the patient (see Additional file 1 for copy of the ANMS GCSI-DD).

Based on the FDA PRO guidance [8], recent FDA guidance on gastroparesis [7], and the FDA feedback to date during the qualification review of the ANMS GCSI-DD, it was necessary to conduct further evaluation of the content validity of the revised ANMS GCSI-DD. The FDA PRO guidance defines content validity by the empiric evidence demonstrating that items and domains of an instrument are appropriate and comprehensive relative to its intended measurement concept, population, and use [8]. Content validity is determined by demonstrating that the structure and content (i.e., items) capture the linkage between the intended measurement concept and the way patients from the population of interest comprehend and discuss the concept [25]. Collecting additional qualitative research provides further support for content validity of the ANMS GCSI-DD as a PRO tool to support a symptom-based endpoint for clinical trials involving diabetic (DG) or idiopathic (IG) gastroparesis patients. Therefore, the primary objective of the current study was to assess the content validity of the ANMS GCSI-DD with patients with DG or IG. The specific aims of the study were to determine if the appropriate symptoms were included in the ANMS GCSI-DD and to evaluate patients’ understanding of the instrument’s instructions, items, response options, and recall period among patients diagnosed with IG or DG.

## Methods

### Study design

To meet the study aims, a cross-sectional qualitative study was conducted that included concept elicitation and cognitive debriefing interviewing components. The study was approved by a central institutional review board (IRB) (Chesapeake; reference number: Pro00022109) and local IRBs at two of the five clinical sites. Regarding sample size, there are no quantitative statistical tests for identifying the minimum numbers of subjects to target for enrollment in qualitative interview studies. In order to determine if sufficient numbers of interviews have taken place to support the results, the goal is to achieve “saturation of concept,” or reaching a point where no new information is forthcoming from the qualitative interview process. Generally, in samples that are relatively homogenous, saturation can be reached with small sample sizes (i.e., 10 patients). Therefore, it was anticipated the target of 25 gastroparesis patients would be sufficient to reach saturation of concept [15, 25, 26, 37].

### Recruitment procedures

Staff members at five clinical sites based in the US were trained by study investigators to identify, screen, recruit, and schedule eligible patients for interviews using a standardized screening script. Site staff identified potential patients through patient databases, medical records, and/or daily appointment schedules. Eligible patients included those who had: clinician-confirmed diagnosis of gastroparesis with documented delay in gastric emptying according to local criteria within the past two years, gastroparesis from either idiopathic or diabetic etiologies, and 18 years of age or older. Patients with gastroparesis from postsurgical causes and/or with lower gastrointestinal disease were excluded. All patients provided informed consent prior to participating in the interview and were remunerated a nominal amount for the time associated with their participation in the study.

### Interview procedures

**Fig. 1:**
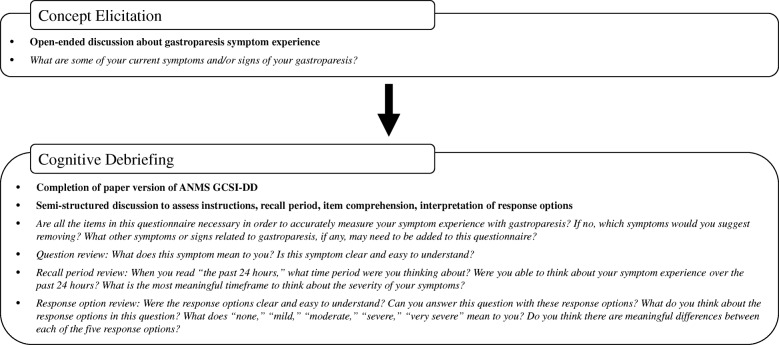
Interview Summary

All interviews were conducted in person and took place at the clinical site locations. The interviews were conducted by three study staff trained in qualitative interviewing methodology, with a combined eight years of experience in conducting qualitative interviews. All interviews were conducted using a semi-structured interview guide with concept elicitation and cognitive debriefing components (Fig. 1).

First, the concept elicitation portion of the interview was conducted to elicit spontaneous descriptions of patients’ symptoms of gastroparesis, and impact(s) of symptoms on their daily lives. Next, patients completed the paper ANMS GCSI-DD and were subsequently engaged in a cognitive debriefing discussion to evaluate the patients’ interpretation of the instructions and items included in the diary, and to ensure that the response options and recall period were appropriate for capturing experiences of patients with IG or DG. Patients were also asked questions about the comprehensiveness, format, and relevance of the content of the ANMS GCSI-DD.

All interviews were conducted in English, took approximately 90 min to complete, and were audio-recorded and subsequently transcribed. Interviewers took detailed notes for each interview. Upon completion of the interview, each patient completed two case report forms (CRFs) that asked questions about patient sociodemographic characteristics and gastrointestinal symptoms in order to characterize the sample of patients in the study. Each clinical site completed a clinical CRF to collect information on relevant medical history of each patient to assist with describing the sample. The total sample size was divided into two rounds of interviews to allow for an interim data analysis in order to determine if changes to the ANMS GCSI-DD were deemed necessary.

### Data analysis

Interview transcripts were assessed with a content analysis approach using ATLAS.ti version 7.5.18 qualitative data analysis software [20]. A coding dictionary was developed, based on the qualitative interview guide, and was supplemented by themes and concepts that emerged from the patient interviews. Using the coding dictionary, two researchers, including those who conducted the interviews, coded the transcripts to identify symptom concepts of gastroparesis and to examine the comprehension of the ANMS GCSI-DD instructions, recall period, items, and response options. A senior team member performed a quality check on a portion of the coded transcripts to ensure that all relevant responses were captured and to ensure that team members were coding consistently. Coded data were organized and summarized into an analysis grid with frequencies and percentages as appropriate. DG and IG patients were analyzed together and separately to determine any similarities and/or differences in their experience of gastroparesis symptoms or interpretation of concepts in the ANMS GCSI-DD.

All quantitative CRF data were entered into a validated study database. Descriptive statistics (mean, standard deviation, frequency, range using SAS version 9.4 [34]) were used to characterize the study sample in terms of sociodemographic and clinical characteristics.

## Results

### Demographic and clinical characteristics

Twenty-five patients were interviewed for this cross-sectional qualitative study conducted from August 2017 – November 2017 across five clinical sites located in California (*n* = 2), New York (*n* = 1), Pennsylvania (*n* = 1), and Massachusetts (*n* = 1). The sites consisted of both small medical clinics and large hospital-based centers.

Of the 25 patients, 15 (60%) were diagnosed with IG and 10 (40%) with DG. The mean age of patients was 42.3 (SD = 14.3; range: 20–70 years), and most of the patients were female (*n* = 19, 76%), white (*n* = 19, 76%), and single/never married (*n* = 12, 48%). Employment and educational status varied across the sample (Table 1).

**Table 1:** Patient Self-reported Sociodemographic Characteristics

Characteristic	Total (*N* = 25)	IG (*N* = 15)	DG (*N* = 10)
Age (years)			
Mean (SD)	42.3 (14.3)	37.9 (13.7)	48.5 (13.2)
Range (Min, Max)	(20, 70)	(20, 70)	(28, 66.0)
Gender, n (%)			
Female	19 (76.0%)	14 (93.3%)	5 (50.0%)
Ethnicity, n (%)			
Not Hispanic or Latino	21 (84.0%)	12 (80.0%)	9 (90.0%)
Racial Background, n (%)^a^			
White	19 (76.0%)	13 (86.7%)	6 (60.0%)
Black or African American	4 (16.0%)	1 (6.7%)	3 (30.0%)
Other^b^	3 (12.0%)	2 (13.3%)	1 (10.0%)
Current Living/Domestic Situation, n (%)			
Living with a partner or spouse, family or friends	18 (72.0%)	4 (26.7%)	3 (30.0%)
Living alone	7 (28.0%)	11 (73.3%)	7 (70.0%)
Current Marital Status, n (%)			
Single/never married	12 (48.0%)	6 (40.0%)	6 (60.0%)
Married or living in marriage-like relationship	9 (36.0%)	7 (46.7%)	2 (20.0%)
Widowed/Separated/Divorced/ Annulled	3 (12.0%)	1 (6.7%)	2 (20.0%)
Other^c^	1 (4.0%)	1 (6.7%)	0 (0.0%)
Employment Status, n (%)^a^			
Employed, full-time	8 (32.0%)	6 (40.0%)	2 (20.0%)
Employed, part-time	5 (20.0%)	1 (6.7%)	4 (40.0%)
Homemaker	2 (8.0%)	2 (13.3%)	0 (0.0%)
Student	1 (4.0%)	1 (6.7%)	0 (0.0%)
Unemployed	1 (4.0%)	1 (6.7%)	0 (0.0%)
Retired	2 (8.0%)	1 (6.7%)	1 (10.0%)
Disabled	9 (36.0%)	5 (33.3%)	4 (40.0%)
Other^d^	1 (4.0%)	0 (0.0%)	1 (10.0%)
Education Status, n (%)			
Secondary/high school/GED	5 (20.0%)	1 (6.7%)	4 (40.0%)
Some college or post-high school education or training	9 (36.0%)	5 (33.3%)	4 (40.0%)
College degree	9 (36.0%)	8 (53.3%)	1 (10.0%)
Postgraduate degree	2 (8.0%)	1 (6.7%)	1 (10.0%)

^a^Not mutually exclusive

^b^Other race: Hispanic, North African, Middle Eastern, Spanish

^c^Other marital status: Engaged

^d^Other employment status: Having four part-time jobs

Clinical characteristics of the sample are displayed in Table 2. On average, patients had been diagnosed with gastroparesis for 1.7 years (range < 1 y to 7 y). Most patients were rated by their clinician as having mild (40%) or moderate (36%) gastroparesis severity levels. All patients (*n* = 25, 100%) were currently receiving treatment for gastroparesis, with 44% on a gastric anti-secretory agent, 36% on a prokinetic agent, and 36% on an antiemetic agent. Most patients (*n* = 21, 84%) had no history of prior GI-specific surgery; the remaining four patients had GI-surgeries unrelated to their gastroparesis.

**Table 2:** Clinician-reported Patient Clinical Characteristics

Characteristic	Total (*N* = 25)	IG (*N* = 15)	DG (*N* = 10)
Time in clinician’s practice (years)			
Mean (SD)	2.7 (3.7)	0.9 (1.2)	5.3 (4.5)
Range (Min, Max)	(0.1, 13.6)	(0.1, 4.2)	(0.5, 13.6)
Duration of diagnosis (years)			
Mean (SD)	1.7 (1.8)	1.7 (2.0)	1.8 (1.6)
Range (Min, Max)	(0.2, 7.0)	(0.2, 7.0)	(0.4, 4.8)
Current medications for gastroparesis, n (%)^a^			
Gastric antisecretory agent	11 (44.0%)	7 (46.7%)	4 (40.0%)
Prokinetic agent	9 (36.0%)	6 (40.0%)	3 (30.0%)
Antiemetic agent	9 (36.0%)	7 (46.7%)	2 (20.0%)
Diabetes medical treatment	7 (28.0%)	0 (0.0%)	7 (70.0%)
Pain medications	5 (20.0%)	5 (33.3%)	0 (0.0%)
Psychotropic agent	4 (16.0%)	3 (20.0%)	1 (10.0%)
Gastric electric stimulation (pacemaker)	1 (4.0%)	0 (0.0%)	1 (10.0%)
Other^b^	4 (16.0%)	4 (26.7%)	0 (0.0%)
Prior GI specific surgeries, n (%)			
Gastric electric stimulation (GES)	1 (4.0%)	0 (0.0%)	1 (10.0%)
Other^c^	3 (12.0%)	2 (13.3%)	1 (10.0%)
None	21 (84.0%)	13 (86.7%)	8 (80.0%)
Time since most recent upper endoscopy (years)			
Mean (SD)	1.2 (1.5)	1.2 (1.6)	1.2 (1.2)
Range (Min, Max)	(0.0, 5.9)	(0.1, 5.9)	(0.0, 3.3)
Physician rating of gastroparesis severity, n (%)			
None	0 (0.0%)	0 (0.0%)	0 (0.0%)
Very mild	3 (12.0%)	1 (6.7%)	2 (20.0%)
Mild	10 (40.0%)	4 (26.7%)	6 (60.0%)
Moderate	9 (36.0%)	7 (46.7%)	2 (20.0%)
Severe	3 (12.0%)	3 (20.0%)	0 (0.0%)
Very severe	0 (0.0%)	0 (0.0%)	0 (0.0%)
Comorbid conditions, n (%)^a^			
Obesity	8 (32.0%)	4 (26.7%)	4 (40.0%)
Other gastrointestinal disorder (other than gastroparesis)^d^	7 (28.0%)	4 (26.7%)	3 (30.0%)
Mood disorder or mental health condition	4 (16.0%)	3 (20.0%)	1 (10.0%)
Insomnia/sleep problems	4 (16.0%)	4 (26.7%)	0 (0.0%)
Respiratory condition	4 (16.0%)	3 (20.0%)	1 (10.0%)
Cardiovascular condition	3 (12.0%)	1 (6.7%)	2 (20.0%)
Dyslipedemia/hyperlipidemia	3 (12.0%)	0 (0.0%)	3 (30.0%)
Neurological condition	1 (4.0%)	0 (0.0%)	1 (10.0%)
Other^d^	4 (16.0%)	3 (20.0%)	1 (10.0%)
None	3 (12.0%)	2 (13.3%)	1 (10.0%)

^a^Not mutually exclusive

^b^Other treatment: Azithromycin, Metoclopramide, Lansoprazole; Botox injection of pyloris; Linaclotide; nasojejunal (NJ) tube, Pantoprazole

^c^Other GI surgery: Cholecystectomy

^d^Other co-morbidity: Fibromyalgia, Iron Deficiency Anemia, Iron Deficiency Anemia, Lyme Disease; Fibromyalgia, Chronic fatigue syndrome, Postural orthostatic tachycardia syndrome (POTS)

### Concept elicitation results

The symptoms most commonly spontaneously reported by patients included: stomach/abdominal pain (*n* = 20, 80% [upper abdominal pain specifically: *n* = 11, 44%]), nausea (*n* = 18, 72%), vomiting (*n* = 13, 52%), bloating (*n* = 11, 44%), early satiety (*n* = 9, 36%), irregular bowel movements (*n* = 9, 36%), and post-prandial fullness (*n* = 6, 24%). Other reported symptoms included acid reflux/regurgitation (*n* = 6, 24%), gas (*n* = 4, 16%), stomach discomfort (*n* = 4, 16%), diarrhea (*n* = 3, 12%), stomach cramps (*n* = 3, 12%), and loss of appetite (*n* = 3, 12%). Table 3 displays representative patient quotes on key symptoms of gastroparesis. Many patients (*n* = 20, 80%) experienced day-to-day symptom change. Gastroparesis symptom concepts did not differ among IG and DG patients.

**Table 3:** Gastroparesis Signs and Symptoms

Symptom	Symptoms Spontaneously ᅟ Reported (N, %)			Representative Quotes
	Total (*N* = 25)	IG (*N* = 15)	DG (*N* = 10)	
Abdominal pain	20 (80%)	13 (87%)	7 (70%)	*“Yeah so for me it’s in my upper left quadrant, like right underneath my rib cage. It’s constant. It’s always there and there’s some days where it’s worse and there’s some days where it’s less and then there’s some days where I also get pain on like my mid-right abdomen.” (005–004)* *“And the abdominal pain that comes either they come in cramps or they come in stabbing pains.” (005–001)*
Nausea	18 (72%)	12 (80%)	6 (60%)	*“I have a low level of nausea all day, like pretty much all day I could throw up and I’m keeping myself from throwing up.” (005–008)*
Vomiting	13 (52%)	8 (53%)	5 (50%)	“*…it’s almost like a fire hydrant sometimes when I go to throw up to where it’s just spewing right out and to where sometimes…I can feel like the muscles straining from trying to open up to get it to come out to where it hurts a lot.” (004–010)* *“Non-stop throwing up for a few minutes, being unable to hold it—the stomach is unable to hold anything in, and even the smell of the breath would be bad. You experience fever, sweat, things like that.” (002–006)*
Bloating	11 (44%)	5 (33%)	6 (60%)	*“It’s just my whole abdomen area, my whole stomach area that pretty much gets bloated…it just get big, like a beer belly.” (002–001)*
Early satiety	9 (36%)	7 (47%)	2 (20%)	*“Feeling full all the time even if I’d only taken a bite or two of food.” (003–001)* *“Not being able to finish half of a meal. It’s frustrating to me not being able to finish um even half of what everybody else is eating.” (004–001)*
Post-prandial fullness	6 (24%)	5 (33%)	1 (10%)	*“Feeling like you can’t possibly put another bite into your mouth.” (002–002)* *“You’re just stuffed full.” (003–001)*

Patients endorsed a variety of different ways that gastroparesis impacts their daily lives, including: physical function (*n* = 17, 68%), social and leisure activities (*n* = 17, 68%), emotional/psychological impacts (*n* = 15, 60%), work/school (*n* = 14, 56%), eating (*n* = 13, 52%), role function (*n* = 5, 20%), relationships with family/friends/significant others (*n* = 5, 20%), and other medical impacts that patients attributed to gastroparesis symptoms (*n* = 11, 44%). Saturation of concepts for symptoms and impacts was achieved.

### ANMS GCSI-DD cognitive debriefing results

Patients were interviewed in two rounds. Eleven patients were interviewed in round one. Based on a review of the interviewer notes from round one patients, no changes to the diary content were deemed necessary. The 14 round two patients were interviewed as a confirmatory cohort. Overall patient feedback on the ANMS GCSI-DD was positive. Patients reported that the diary was quick to complete, contained a reasonable number of questions, and was easy and straightforward to comprehend.

#### Instructions

Overall, patients demonstrated a clear understanding of the instructions for completing the nausea, early satiety, post-prandial fullness, and upper abdominal pain severity items: “*These questions ask about symptoms you may have each day. Please complete the daily diary at about the same time every evening. For each symptom listed below, please* *mark with an X the box* *that best describes the worst severity* *of each symptom* *during the past 24 hours*. *Please be sure to answer each question.”*

When 23 patients were asked specifically if they thought about the worst experience, 19 patients (83%) correctly reported that they considered each symptom at its worst throughout the past 24 h. The remaining four patients (17%) responded that they thought about their symptoms as an average of the past 24 h, but noted that their answers would not change if they thought instead about their worst experience.

Item 5 (vomiting) had a separate set of instructions specific for vomiting that read: “*The next question asks you to record the number of times vomiting occurred in the last 24 hours. Please record the number of vomits (throwing up with food or liquid coming out) that occurred in the last 24 hours. Record zero, if you have not vomited during the past 24 hours. If you vomited, write down the number of all vomits. If you vomited once, record one. If you vomited three times during the day, record three. If you vomited three times, whether it was during the same trip to the bathroom or three separate trips, record three as the number of episodes of vomiting.”* The majority of patients (*n* = 23, 92%) demonstrated a clear understanding of the instructions and correctly explained how they should be counting the number of vomiting episodes. The remaining patients (*n* = 2, 8%) stated that they should count the number of trips to the bathroom instead of counting the number of vomiting episodes. However, these two patients reported 30 or more vomiting episodes per day.

#### Recall period

There were no comprehension issues related to the 24-h recall period, and all 25 patients (100%) correctly reported that they considered their experiences over the past 24 h when answering the questions. Most patients stated that the most meaningful timeframe to think about the severity of their symptoms was the past 24 h (*n* = 15/24, 63%). Other patients suggested that the diary could ask about the past seven days (*n* = 4/24, 17%), previous night into the next morning (*n* = 1/24, 4%), only at the end of the day (*n* = 1/24, 4%), only in the morning (*n* = 1/24, 4%), the past month (*n* = 1/24, 4%), or before a gastroparesis procedure (*n* = 1/24, 4%).

#### Response options

The ANMS GCSI-DD utilizes a 5-point verbal rating scale for the nausea, early-satiety, post-prandial fullness, and upper abdominal pain items. The response options for the symptom severity items include none, mild, moderate, severe, and very severe. The vomiting frequency item has an open-ended field that allows for numerical responses of vomiting episodes, with a lower range of 0. All the patients had a clear understanding of both response scales and were able to easily select a response and describe how they arrived at their answers. Additionally, most patients (*n* = 19/25, 76%) endorsed meaningful changes between each response option. However, three patients (12%) found “very severe” and “severe” to mean the same thing, and an additional three patients (12%) reported no meaningful difference between “mild” and “moderate” response options.

#### Item relevance and importance

Patients endorsed the gastroparesis signs and symptoms included in the ANMS GCSI-DD as relevant and/or important to their gastroparesis experience, as follows: early satiety (*n* = 25, 100%), post-prandial fullness (*n* = 25, 100%), nausea (*n* = 22, 88%), upper abdominal pain (*n* = 18, 72%), and vomiting (*n* = 15, 60%).

Many patients (*n* = 11, 44%) reported that all relevant gastroparesis symptoms were captured within the ANMS GCSI-DD. However, some patients suggested that the diary could benefit from additional questions about other symptoms. These suggestions included symptoms related to bowel movements (i.e., frequency, consistency, diarrhea, constipation [*n* = 7, 28%]), and bloating (*n* = 4, 16%). The following were endorsed by one patient (not necessarily the same patient): discomfort, slow digestion, feeling of an empty stomach, difficulty breathing, acid reflux, dry heaving, and impaired concentration.

#### Item level feedback

The item level feedback is displayed in Table 4. Specifically, the results on patient definition and understanding of the items, their description of the items, alternative or qualifying feedback on the items, and additional comments are reported.

**Table 4:** Item Level Cognitive Debriefing

ANMS GCSI-DD Item	Patient Clearly Defined and Demonstrated Understanding	Patient Description	Alternatives or Qualifiers	Other Comments
Item 1: Nausea (feeling sick to your stomach as if you were going to vomit or throw up)	*n* = 25, 100%	Patient “feeling sick to their stomach,” “feeling like they will throw up,” “sickness in the stomach,” “upset stomach,” and “queasy.”	One patient (4%) explained how she experiences different types of nausea and at times can experience nausea without feeling like she will throw up.	All patients thought the phrase in parentheses (“feeling sick to your stomach as if you were going to vomit or throw up”) was a clear and appropriate definition for nausea.
Item 2: Not able to finish a normal-sized meal (for a healthy person)	*n* = 24, 96%	Patient unable to eat a normal-sized meal, unable to finish a normal plate of food, a remature loss of appetite, or “getting full quickly.”	Patients explained that a regular meal for them would entail a half-sized portion of a “normal” meal such as half a cup of soup, half a sandwich, or half of a burger.	Patients correctly defined a normal-sized meal “for a healthy person,” explaining that it would entail a “well-balanced meal” consisting of “multiple servings or courses” or the “right” amount of protein, carbohydrates, vegetables, and fruit or dessert.
Item 3: Feeling excessively full after meals	*n* = 25, 100%	Patient terms such as “stuffed” or feeling like there is “too much food” in their stomach.	All but one patient (*n* = 24, 96%) successfully described the difference between Item 2 (early satiety) and Item 3 (post-prandial fullness).	No other comments
Item 4: Upper abdominal pain (above the navel)	*n* = 25, 100%	Patient pain above the navel/belly button or near the middle of the stomach and further described the feeling of pain as “sharp,” “deep,” “dull,” “burning,” and “uncomfortable.”	Most patients (*n* = 12, 48%) endorsed “upper abdominal pain (above the navel)”, as appropriate to capture their symptoms, while others preferred the term “abdominal pain” (*n* = 9, 36%) or did not have a preference (*n* = 2, 8%).	All patients expressed understanding of the term “navel.”
Item 5: During the past 24 h, how many episodes of vomiting did you have?	*n* = 25, 100%	Patient “episode” of vomiting as food or liquid being expelled from the mouth.	Most patients (*n* = 15, 60%) agreed that it was important to assess the frequency of vomiting, while others suggested that the item could assess the severity of vomiting (*n* = 3, 12%) or ask about retching and dry heaving (*n* = 3, 12%) as an indicator of symptom severity.	All patients demonstrated they could answer the vomiting item using the written response option space, and most patients (*n* = 23, 92%) provided a clear explanation for how to count their episodes of vomiting over the past 24 h. Two patients (8%) reported that they would experience some difficulty with counting their episodes of vomiting over the past 24 h due to high frequency of vomiting episodes.

## Discussion

Overall, patients provided positive feedback on the ANMS GCSI-DD, and reported that the instrument was straightforward, comprehensive, and relevant to their experience with gastroparesis. In addition, the instructions, item wording, and response options were noted to be well-understood by the majority of patients. Patients demonstrated that they could easily select a response option and describe how they arrived at their answers. The symptoms included in the ANMS GCSI-DD reflected the symptom experiences of patients with gastroparesis. Additionally, no significant differences in symptom experience were observed between patients diagnosed with DG or IG.

Some suggestions and recommendations were made by patients that require some reflection and discussion. First, bloating was endorsed by 11 (44%) patients as a relevant symptom of gastroparesis, four of which (16%) suggested adding this symptom to the diary. It should be noted that bloating was part of the original GCSI measure but was subsequently removed following FDA recommendations and previous research that indicated overlap in postprandial fullness and bloating concepts by gastroparesis patients [30]. Furthermore, the current study showed that less than half of patients endorsed bloating. Future clinical studies may include the bloating item as an exploratory symptom for gastroparesis.

Additionally, seven patients (28%) suggested that the ANMS GSCI-DD could be improved upon by adding a question about bowel movements (i.e., frequency, consistency, diarrhea, constipation). These recommendations are consistent with previous qualitative research on the GCSI and GCSI-DD [30]. However, bowel movement-related symptoms are not directly associated with the clinical manifestations of gastroparesis and may reflect other gastrointestinal comorbidities concurrent with gastroparesis in the study patients [18].

Regarding the overall instructions of the ANMS GCSI-DD, there were four patients (17%) who inadvertently considered the average of their symptom severity over the past 24 h as opposed to the worst severity. However, these patients mentioned that their answers would not have changed had they considered the worst severity instead of the average rating. The majority of patients (83%) understood the instruction meaning as intended, therefore no content changes are recommended to the instructions.

For Item 4, Upper Abdominal Pain (above the navel), patients were asked which term, “upper abdominal pain (above the navel)” or “abdominal pain” best captured their gastroparesis symptoms. Most patients (*n* = 12, 48%) endorsed “upper abdominal pain (above the navel)”, while others preferred the term “abdominal pain” (*n* = 9, 36%) or had no preference (*n* = 2, 8%). Since there was no consensus, and since the majority of the sample (96%) demonstrated that they were able to differentiate among upper, lower, and overall abdominal pain, no content changes to the item are supported.

For Item 5, Vomiting, an episode of vomiting was well understood by the majority of patients (*n* = 23, 92%). However, two patients (8%) found it difficult to count the number of vomiting episodes in 24 h due to experiencing a high frequency of episodes. From a scoring and weighting standpoint, there are no major issues since the ceiling for the scale is four or more episodes of vomiting and adequately captures this increased frequency of vomiting. It should also be noted that 48% of the patient sample did not experience issues with vomiting from gastroparesis, which may be related to the severity of their current gastroparesis condition [23].

The severity response scale used for the ANMS GCSI-DD is consistent with the recommended response scale in the FDA guidance on gastroparesis outcomes and endpoints [7]. Regarding the severity response options (None/Mild/Moderate/Severe/Very Severe), the majority of patients demonstrated a good understanding of and endorsed a meaningful difference between each response option. A few patients expressed that some of the response options were the same or very similar (e.g., mild and moderate, severe and very severe) and/or that they would consider a 2-point jump as a meaningful improvement (e.g., very severe to moderate) as opposed to a 1-point jump. However, no changes to the scale are recommended since the scale was well understood by the majority of patients and most patients interpreted a 1-point jump as meaningful improvement on the scale. In addition, previous item response theory analyses of the ANMS GCSI-DD with this response scale demonstrated good model fit and ordered response categories [31]. Guidelines for cognitive interviewing for new PROs recommend evaluating respondent understanding of the response scales [26, 37] consistent with the procedures reported in this study.

A strength of the study was that it included 25 gastroparesis patients recruited from geographically diverse regions of the US and from both small and large medical clinics. Although the sample was predominantly white (76%), patients from different ethnicities were represented in the research. Study limitations included reliance on patient self-report and recall bias. The study sample may also be biased toward patients with less severe disease, as patients with severe gastroparesis may have been less likely to agree to participate in the research study.

Additional research is in progress to generate quantitative evidence on the psychometric measurement properties (e.g., reliability, validity) needed to support full FDA qualification of the ANMS GCSI-DD. Translations and migration to an electronic handheld device are also being implemented to facilitate future use of the instrument in multi-national clinical trial studies and clinical settings.

## Conclusions

In summary, the evidence obtained from this study provides strong support for the content validity and patient understanding of the ANMS GCSI-DD for use as a PRO tool to support a symptom-based clinical trial endpoint to evaluate the treatment effect in both IG and DG patient populations. The symptoms included in the ANMS GCSI-DD are important, relevant, and well understood among patients diagnosed with IG or DG. The ANMS GCSI-DD may represent an acceptable symptom outcome measure for evaluating the efficacy of new gastroparesis treatments. Before confirmation of the ANMS GCSI-DD as a primary or key secondary outcome for clinical trials, evidence needs to be generated on reliability, validity, and sensitivity to changes in clinical status. In addition, inclusion of the daily diary in phase 2 clinical trials as a secondary or exploratory endpoint may be warranted.

## Additional file

Additional file 1: The ANMS GCSI-DD version used in the study reported herein is paper-based, uses a 24-hour recall period, and consists of five symptoms: nausea, early satiety, post-prandial fullness, upper abdominal pain, and vomiting. Nausea, early satiety, post-prandial fullness, and upper abdominal pain are rated using a 5-point verbal rating scale, and the number of vomiting episodes over the last 24 hours is recorded by the patient. (DOCX 14 kb)

## Abbreviations

ANMS GCSI-DD: American Neurogastroenterology and Motility Society Gastroparesis Cardinal Symptom Index-Daily Diary
CRFs: Case report forms
DG: Diabetic gastroparesis
FDA: Food and Drug Administration
GCSI: Gastroparesis Cardinal Symptom Index
GE: Gastric emptying
HRQL: Health-related quality of life
IG: Idiopathic gastroparesis
IRB: Institutional review board
PAGI-SYM: Patient Assessment of Upper Gastrointestinal Disorders-Symptoms
PRO: Patient-reported outcome
US: United States
