# Supplementary material for: The content validity of the ANMS GCSI-DD in patients with idiopathic or diabetic gastroparesis
Source: J Patient Rep Outcomes. 2018 Dec 13;2:61. doi: 10.1186/s41687-018-0081-2 (PMC6292831; doi:10.1186/s41687-018-0081-2)
Supplement: Supplementary file 1 — The ANMS GCSI-DD version used in the study reported herein is paper-based, uses a 24-hour recall period, and consists of five symptoms: nausea, early satiety, post-prandial fullness, upper abdominal pain, and vomiting. Nausea, early satiety, post-prandial fullness, and upper abdominal pain are rated using a 5-point verbal rating scale, and the number of vomiting episodes over the last 24 hours is recorded by the patient. (DOCX 14 kb) [file 41687_2018_81_MOESM1_ESM.docx]

# **Additional Files**

Copy of the American Neurogastroenterology and Motility Society Gastroparesis Cardinal Symptom Index-Daily Diary (ANMS GCSI-DD) Instrument

**ANMS GASTROPARESIS CARDINAL SYMPTOM INDEX – DAILY DIARY**

| **Instructions:** These questions ask about symptoms you may have each day. Please complete the daily diary at about the same time every evening. | | | | | | |
| --- | --- | --- | --- | --- | --- | --- |
| For each symptom listed below, please mark with an X the box that best describes the worst severity of each symptom during the past 24 hours. Please be sure to answer each question. | | | | | | |
|  | | **None** | **Mild** | **Moderate** | **Severe** | **Very Severe** |
| 1. | Nausea (feeling sick to your stomach as if you were going to vomit or throw up) | □ | □ | □ | □ | □ |
| 2. | Not able to finish a normal-sized meal (for a healthy person) | □ | □ | □ | □ | □ |
| 3. | Feeling excessively full after meals. | □ | □ | □ | □ | □ |
| 4. | Upper abdominal pain (above the navel). | □ | □ | □ | □ | □ |

The next question asks you to record the number of times vomiting occurred in the last 24 hours. Please record the number of vomits (throwing up with food or liquid coming out) that occurred in the last 24 hours. Record zero, if you have not vomited during the past 24 hours. If you vomited, write down the number of all vomits. If you vomited once, record one. If you vomited three times during the day, record three. If you vomited three times, whether it was during the same trip to the bathroom or three separate trips, record three as the number of episodes of vomiting.

5. During the past 24 hours, how many episodes of vomiting did you have? ____
